# Clinical severity and high-resolution CT severity score in COVID-19: Is there an association

**DOI:** 10.12669/pjms.40.4.7919

**Published:** 2024

**Authors:** Raja Mobeen Ahmed, Kaleem Ullah Toori, Muhammad Arsalan Qureshi

**Affiliations:** 1 Raja Mobeen Ahmed, MBBS. Department of Medicine, KRL Hospital, Islamabad, Pakistan; 2 Kaleem Ullah Toori, FRCP. Department of Medicine, KRL Hospital, Islamabad, Pakistan; 3 Muhammad Arsalan Qureshi, MBBS. Department of Medicine, KRL Hospital, Islamabad, Pakistan

**Keywords:** COVID-19, CT-severity score, High resolution CT, Mortality, Comorbids, Inflammatory Markers, Disease severity

## Abstract

**Objectives:**

To identify a correlation between the clinical parameters and CT chest severity score in COVID-19.

**Methods:**

A total of 205 RT-PCR positive patients were included in this descriptive cross-sectional study with convenience sampling from November 2020 to June 2021 in KRL Hospital. The study population was stratified in disease severity as per the WHO’s guidelines. Clinical and radiological characteristics were compared in survivors and non survivors to draw conclusion

**Results:**

The mean age was 57 years and the majority of the patients 57% were male. Overall mortality was 22% and the mean CT severity score was 18. Non survivors were more tachypneic, hypoxic, had a higher CT chest severity score, higher clinical severity, more comorbid condition and higher TLC, D-Dimers, LDH, CRP, NLR. Raised CT severity score showed a conclusive correlation with greater disease severity. One way ANOVA showed a significant difference between mean CT severity score amongst different disease categories.

**Conclusion:**

Higher CT severity score corresponds to a higher clinical severity and higher chances of mortality.

## INTRODUCTION

The COVID-19 has been found to have certain radiological and clinical characteristics, some of which are unique and in the right clinical context, can be pathognomonic of the former. Well over 400 million people have been affected with the disease so far.^1^,^2^ Reverse Transcriptase Polymerase chain reaction [RT-PCR] detection is the gold standard for diagnosing COVID-19 when clinically suspected.^3^,^4^ Given the significant false negative rate due to incorrect sampling technique or time since symptoms, and a high number of patients the facility of RT-PCR is not readily available and every patient may not be amenable to RT-PCR testing resources.

High-resolution computed tomography (HRCT) of chest has been used as a means to recognize such high-risk patients noninvasively and quickly.^4^ Based on High-resolution computed tomography, the COVID-19 reporting and data system classification (CO-RADS) and Chest CT severity score (CTSS) have been devised and validated as markers of lung involvement in patients affected by SARS-COV-2.^5^,^6^ To further ease the diagnosis; WHO has outlined the typical patterns of lung involvement on high resolution computed tomography which can help differentiate COVID -19 from other conditions.^7^ Also CTSS score may help identify patients with clinically severe disease who would need early intervention.^7^,^8^

The disease pattern of COVID-19 has been different in various racial and geographical distributions depicting different incidence and mortality rates, as reported in the literature, since the start and through various waves of the pandemic.^9^,^10^ In this study, we aimed to identify if there is a correlation between clinical severity parameters and CT Chest severity scores in COVID-19 patients in our local population, so as to use this tool in triaging potentially high-risk patients needing prompt treatment and care.

## METHODS

The study was approved by local Ethical review committee letter vide “Ref ERC: KRL-HI-ERC/2020/24-B” dated 16-11-2020.The Study design was descriptive, cross sectional with non-probability convenience sampling and the patients were recruited after informed consent. All patients over 18 years with signs and symptoms of COVID -19 who presented to the fever clinic whom had a positive RT-PCR result for COVID -19, were included. Exclusion Criteria was anyone with a negative RT-PCR.

### Ethical Approval

This study was conducted at KRL hospital in Islamabad and included patients with COVID -19 admitted to a dedicated COVID-19 ward or ICU, from November 2020 to June 2021.

The patients were categorized using the WHO defined parameters of Clinical Severity^11^ at the time of presentation to the fever clinic as follows:

- Non-Severe disease - Being PCR +ve, typical signs and symptoms of COVID -19 without presence of signs of Severe and Critical disease.
- Severe - in addition to above, Oxygen saturation < 90% on room air, Respiratory rate > 30 breaths/min or signs of severe respiratory distress (accessory muscle use, inability to complete full sentences).
- Critical - presence of acute respiratory distress syndrome (ARDS), sepsis or septic shock in patients with severe COVID-19 infection.

Radiological severity was measured using the validated CT chest severity score; which is the sum of the score of the 20 lung regions which are further scored as per their involvement of the parenchymal opacification. Scores are given as 0 for no involvement of lung segment, one for less than 50% lung involvement and two for more than 50% lung involvement. The total score is the sum of all the segments involved ranging from 0-40; to score the extent of lung involvement in COVID-19 patients.^6^ Radiological severity was defined as per the CT chest severity score, into non-severe and severe disease with a cutoff of 19.5. The CT scans were reviewed and reported by a classified consultant radiologist as part of the clinical care as per Hospital’s protocol.

Patient’s sign and symptoms, demographic profile, and comorbid conditions were recorded**.** Baseline labs including Blood complete picture, Liver Functions test, Renal Function tests, Coagulation profile-reactive protein, Lactate Dehydrogenase, D-dimers, Serum Ferritin, were sent as per hospital’s COVID protocol. The admitted patients were followed till discharge or death. The non-admitted patients were followed in outpatient basis with weekly clinic assessment for two weeks or until recovery/ PCR was negative as per the hospital’s protocol.

Pearson Chi squared testing was used to determine association between categorical variables with significance of p< 0.05 and confidence interval of 95%. CT chest severity and Clinical Severity groups were analyzed separately. Independent T test was used to determine association between the CT severity groups and the lab parameters. To determine difference amongst the means of the Clinical severity and CT chest severity scores, one way ANOVA was used, and Tukey’s HSD was used to check the differences amongst specific group means. Data was analyzed using SPSS-26.

## RESULTS

A total of 205 patients were recruited. The mean age was 57±14.2 years with 57% (n= 117) males, 62.4% (n=128) patients required hospitalization out of whom 22.9% (n=47) died. Amongst clinical severity sub-groups, 48.3% (n=99) had non-severe disease, 35.1% (n=72) had severe disease and 16.6 % (n=34) had critical disease. The mean CT-SS was 18, Inter Quartile Range (10-22). Radiological severity was severe in 43.4% (n= 89) patients. (Table-I) The most common symptoms were fever 48.8% (n=183), cough 69.3% (n= 92) and shortness of breath 59.5% (n=122). The median duration of symptoms was six days. (Table-I) The CT chest severity score was initially analyzed ungrouped with all the variables as mentioned under the heading “Combined P value” (Table-I) and it was also analyzed in two groups stratified by the score of 19.5 as mentioned in the CT-Chest severity score study.

**Table-I:** Showing a Comparison of Demographic Data, Vital signs and Laboratory Investigations between CT-Severity and Clinical Severity Groups.

Parameters	CT-Severity score				Clinical Severity		

	<19.5	>19.5	Total	P values	Non-Severe	Severe and Critical	(Combined) P values
N	116(56.6)	89(43.4)	205	-	99	106	
CT-SS**	11 (7-16)	23 (21-29)	18 (10-22)		13.9	20.7	0.017
Age	55.0±14.3	60.8±13.4	57.5±14.2	0.004	52.98± 14.7	61.77±12.2	0.319
Male	64 (55.2)	53 (59.6)	117 (57.1)	-	54(54.5)	63(59.4)	-
Admission	61 (52.6)	67 (75.3)	128 (62.4)	-	25(25.2)	103(97.1)	-
Death	14 (12.1)	33 (37.1)	47 (22.9)	<0.001	3(0.03)	44(41.5)	<0.001
Symptom Duration**	6 (3-9)	6 (4-10)	6 (4-9)	0.420	6.19	7.17	0.424
Hypertension	55 (47.4)	45 (50.6)	100 (48.8	0.020	40	60	0.020
Diabetes	47 (40.5)	45 (50.6)	92 (44.9)	0.152	41	51	0.335
CKD	10 (8.6)	7 (7.9)	17 (8.3)	0.005	10	07	0.364
CLD	3 (2.6)	2 (2.2)	5 (2.4)	0.876	02	03	0.707.
IHD	15 (12.9)	14 (15.7)	29 (14.1)	0.569	14	15	0.998
Respiratory disease	10 (8.6)	8 (9.0)	18 (8.8)	0.926	07	11	0.403
Neurological disease	3 (2.6)	4 (4.5)	7 (3.4)	0.043	03	04	0.770
Cancer	4 (3.4)	0 (0.0)	4 (2.0)	0.077	01	03	0.346
Number of Comorbidities	-	-	-	0.010	-	-	0.016
0	45 (38.8)	20 (22.5)	65 (31.7)	-	41	24	-
1	21 (18.1)	30 (33.7)	51 (24.9)	-	51 (24.9)	30	-
2	50 (43.1)	39 (43.8)	89 (43.4)	-	37	52	-
Blood pressure	-	-	-	0.148	-	-	0.004
Abnormal	27 (23.3)	36 (40.4)	63 (30.7)	-	21	42	-
Heart Rate**	90 (81-100)	92 (86-104	90 (84-102)	0.143	90.95(80-100)	95.64(85-104)	0.330
Respiratory rate /min**	21 (19-24)	24 (20-28)	22 (20-26)	0.085	20.79(18-21)	25.67(22-28)	<0.001
Temperature F**	98.6 (98.6-100.0)	98.6 (98.6-99.0)	98.6 (98.6-100.0)	0.002	99.13(98.6-100.0)	99.10(98.6-100.0)	0.236
Oxygen saturation	94 (90-96)	90 (82-93)	92 (88-95)	<0.001	94(94-96)	84(82-90)	<0.001
Severity	-	-	-	<0.001	-	-	
Non-severe	72 (62.1)	27 (30.3)	99 (48.3)	-	-	-	-
severe	36 (31.0)	36 (40.4)	72 (35.1)	-	-	-	-
Critical	8 (6.9)	26 (29.2)	34 (16.6)				
Hemoglobin**	12.9 (11.8-14.1)	13.3 (12.2-14.7)	13.1 (11.9-14.3	0.446	13.1(12.1-14-4)	12.9(11.7-14.2)	0.422
Platelets**	198 (150-280)	201 (129-256)	198 (149-264)	0.334	218(150-271)	210(145-257)	0.538
TLC**	8.2 (6.3-10.9)	8.7 (6.7-11.3)	8.4 (6.4-11.0)	0.002	8.8(6.1-10.4)	9.7(6.7-11.7)	0.106
Lymphocytes**	19 (10-28)	12 (7-20)	15 (8-24)	0.608	21.2(10-28)	13.8(7-19)	<0.001
Neutrophils**	74 (64-83)	80 (74-87)	78 (66-85)	0.064	70.0(62-82)	78.7(73.7-88)	<0.001
NLR	4.0 (2.3-8.7)	6.9 (4.0-12.6)	5.4 (2.8-10.6)	0.555	6.2(2.2-7.8)	10.1(3.9-13.4)	0.002
Lymphopenia	83 (71.6)	78 (87.6)	161 (78.5)	0.449	65	96	<0.001
CRP**	50.0 (13.0-104.8)	104.0 (40.7-135.3)	65.0 (24.9-131.5)	0.830	75.3(13.4-97.1)	103.9(43.3-150)	0.014
D-Dimers**	422 (206-710)	575 (333-1379)	474 (273-987	0.028	1027(205-768)	1219(320-1145)	0.462
Ferritin**	394 (207-740)	648 (430-1008)	544 (256-894)	0.254	678(196-659)	896(381-1096)	0.126
LDH**	302 (238-405)	409 (298-538)	343 (250-475)	0.005	336(237-411)	419(277-541)	0.001

* Mean ±SD,

** Median (Interquartile Range).

Overall, by comparing the CT group and Clinical severity groups, mortality was found to be statistically significant across both groups (p=<0.001). The independent t test was run to see the correlation between non-severe (M=13.92, SD 7.30) and severe disease (M= 20.10; SD 8.73) with CT chest severity scores. Additionally, the homogeneity of Levene’s statistic was satisfied F (2.0) p=(0.157). The independent t-test was associated with statistically significant effect t (-5.50) = 200, p<0.001. This shows that severe disease is associated with a higher CT-chest severity score.

The mean CT-SS showed a rising trend signifying a positive correlation with clinical severity, (Table-II). To further explore the difference in mean CTSS across different clinical severity groups, analysis via Tukey’s HSD test showed significant mean difference across all categories (p value < 0.05; Table-III) one-way ANOVA indicated a significant difference amongst means of CT-SS between and within groups in our study population (*F value* (2,202) = 24.7, p *value* <0.001); inferring a significant difference of mean CT-SS between non-severe and severe disease categories.

**Table-II:** Mean CTSS score and Clinical severity at 95% confidence interval for Mean.

Clinical Severity	N	Mean CTSS	Std. Deviation	Std. Error	Lower Bound	Upper Bound	Minimum	Maximum
Non-Severe	99	13.91	7.30	0.733	12.45	15.36	1	33
Severe	72	17.92	8.148	0.960	16.00	19.83	2	37
Critically ill	34	24.62	8.265	1.417	21.73	27.50	4	36

Total	205	17.09	8.621	0.602	15.91	18.28	1	37

**Table-III:** Tukey HSD.

Clinical Severity	Clinical Severity	Mean Difference	Std. Error	P value	Lower Bound	Upper Bound
Non-severe disease	Severe	-4.008	1.203	0.003	-6.85	-1.17
	Critical Disease	-10.709	1.544	0.000	-14.35	-7.06
Severe disease	Non-severe disease	4.008	1.203	0.003	1.17	6.85
	Critical Disease	-6.701	1.616	0.000	-10.52	-2.89
Critical Disease	Non-severe disease	10.709	1.544	0.000	7.06	14.35
	Severe disease	6.701	1.616	0.000	2.89	10.52

## DISCUSSION

We found a positive association between mortality from COVID-19 and age; as the age increase the mortality was higher as well. The presence of comorbid conditions including hypertension, chronic kidney disease and chronic neurological disorders, low oxygen saturation, and high inflammatory markers and the total of co morbid conditions was correlated with having a higher CT-chest severity score which was in turn linked to having a higher chance of mortality. An Indian study which showed middle aged men, presence of underlying comorbidities, and hypertension being associated with higher risk of mortality.^12^

Furthermore, low oxygen saturation is an independent risk factor for mortality. As we already are aware that advancing age leads to sub normal immune response^13^, we infer that the population with higher age may be at a risk for having poor outcomes. We found no correlation of gender with mortality. CT chest severity score has been used to quantify the severity of lung disease with COVID-19; the higher scores correspond to higher disease burden and has been reported by Hu Y et al^14^ and also by local tertiary care centers.^15^,^16^

Our results are in line with this finding. There is evidence from literature review that severity of COVID-19 has also been associated with presence of high inflammatory markers. This study revealed significant relationship between a higher CT chest severity score and Lymphopenia, high D-Dimers, low Ferritin and high LDH. This corresponds with studies across the globe reporting severe COVID-19 on CTSS and higher values of CRP, LDH, D-dimers, Ferritin and worsening lymphopenia, including one of our own previous publications^17^, thus further validating our results.^18^-^20^ Studies across Pakistan have also reported high inflammatory markers with higher severity of the disease which in turn increase the chances of developing complications and were associated with a higher mortality.^21^ Francone reported no relationship between CT severity score and severe and critical disease groups; it may be attributed to small sample size.^22^

### Limitations & Strength of the study

First, it was a single center study, but included diverse population group from all ages and fairly distributed comorbidities. Second, dynamic CT changes were not included but this actually might be a strength of our study in terms of signifying use of CT rather than dynamic CT, given latter is not easily accessible or readily available at most hospitals. The foremost strength of the study was the inclusion of only PCR positive patients who underwent advanced imaging to see the radiological severity.

## CONCLUSION

A higher CT severity score was related to a higher clinical severity which was associated with a poor outcome. Identifying high-risk patients with COVID-19 is crucial to utilize resources efficiently in their management leading to a better prognosis.

### Author`s Contribution:

**RMA** conceived, designed did statistical analysis along with review of manuscript.

**KT** contributed to data collection and final review of manuscript.

**MAQ** did manuscript writing and data collection.

**KUT** is responsible for the integrity and accuracy of the work.
